# Effects of Different Cultivation Modes on Morphological Traits and Correlations between Traits and Body Mass of Crayfish (*Procambarus clarkii*)

**DOI:** 10.3390/biology13060395

**Published:** 2024-05-30

**Authors:** Jinlong Li, Qin Qin, Xing Tian, Jiarong Guo, Bowen Tang, Zhigang He, Zhonggui Xie, Yude Wang, Dongwu Wang

**Affiliations:** 1 Hunan Fisheries Science Institute, Changsha 410022, China; 2 Fisheries College, Hunan Agricultural University, Changsha 410125, China; 3 State Key Laboratory of Developmental Biology of Freshwater Fish, College of Life Sciences, Hunan Normal University, Changsha 410081, China

**Keywords:** body weight, cultivation mode, gender, morphological traits, *Procambarus clarkii*

## Abstract

**Simple Summary:**

It is well known that animal morphological traits are influenced by both genetic and environmental factors, but further research is needed to investigate the extent to which the environment influences morphological traits under the same genetic background. As one of the most widely distributed freshwater shrimp species in the world, the crayfish has a strong territorial and combative habit, so the influence of the aquaculture environment on its morphological development is more obvious. Meanwhile, under the same environment, different sexes of crayfish also showed corresponding morphological differences due to their reproductive functions. In this study, juvenile crayfish hatched from the same population were cultured in different growing environments, and the effects of environment and sex on their morphological characteristics were evaluated by mathematical models such as correlation analysis, pathway analysis, and gray correlation, so as to provide a basis for the artificial selection of crayfish in the later stage of development.

**Abstract:**

In this study, juvenile crayfish hatched from the same population were cultured in different growing environments: pond (***D*1**), paddy field (***D*2**), and aquaculture barrel (***D*3**), and fed for 60 days. Crayfishes were selected randomly, females and males, 50 tails each from six groups (D1-♀, D1-♂, D2-♀, D2-♂, D3-♀, D3-♂) to measure the following morphological traits: full length (*X*_1_), body length (*X*_2_), chelicerae length (*X*_3_), chelicerae weight (*X*_4_), cephalothorax length (*X*_5_), cephalothorax width (*X*_6_), cephalothorax height (*X*_7_), eye spacing (*X*_8_), caudal peduncle length (*X*_9_), and caudal peduncle weight (*X*_10_). We found that the coefficient of variation (CV) of *X*_4_ was the largest in each culture mode, and males (28.58%~38.67%) were larger than females (37.76%~66.74%). The CV of *X*_4_ of crayfish cultured in ***D*1** and ***D*2** was larger than that of ***D*3**. All traits except *X*_8_ were positively correlated with body weight (*p* < 0.05). After pathway analysis, we found that *X*_4_, *X*_5_, *X*_7_, and *X*_10_ were significantly correlated with the body weight of D1-♀; the equation was *Y*_D1-♀_ = −29.803 + 1.249*X*_4_ + 0.505*X*_5_ + 0.701*X*_7_ + 1.483*X*_10_ (*R*^2^ = 0.947). However, *X*_2_, *X*_4_, and *X*_6_ were significantly correlated with the body weight of D1-♂; the equation was *Y*_D1-♂_ = −40.881 + 0.39*X*_2_ + 0.845*X*_4_ + 1.142*X*_6_ (*R*^2^ = 0.927). In D2-♀, *X*_1_, *X*_4_, *X*_5_, and *X*_10_ were significantly correlated with body weight; the equation was *Y*_D2-♀_ = −12.248 + 0.088*X*_1_ + 1.098*X*_4_ + 0.275*X*_5_ + 0.904*X*_10_ (*R*^2^ = 0.977). *X*_4_ and *X*_5_ played a major role in the body weight of D2-♂ with the equation: *Y*_D2-♂_ = −24.871 + 1.177*X*_4_ + 0.902*X*_5_ (*R*^2^ = 0.973). *X*_3_ and *X*_10_ mainly contributed to the body weight of D3-♀ with the equation: *Y*_D3-♀_ = −22.476 + 0.432*X*_3_ + 3.153*X*_10_ (*R*^2^ = 0.976). *X*_1_ and *X*_4_ mainly contributed to the body weight of D3-♂ with the equation: *Y*_D3-♂_ = −34.434 + 0.363*X*_1_ + 0.669*X*_4_ (*R*^2^ = 0.918). Comparing the pathway analysis with the gray relation analysis, we could conclude that the traits most correlated with body weight in D1-♀ were *X*_10_ and *X*_7_; in D1-♂, *X*_6_; in D2-♀, *X*_10_, *X*_1_, and *X*_5_; in D2-♂, *X*_5_; in D3-♀, *X*_10_; and in D3-♂, *X*_4_ and *X*_1_.

## 1. Introduction

It is well known that morphological traits are affected not only by genetic [1], gender [2], and other innate factors [3], but also by the growing environment [4]. Individuals of the same species can display high phenotypic variation often in response to varying environmental conditions. Dani et al. compared the morphological traits of the captive population with the wild population of the endangered Spanish toothcarp (*Aphanius iberus*) [5], and showed significant differences in morphological features between populations and sexes. Noëlle et al. found that different breeding environmental conditions could affect the body shape of the pumpkinseed (*Lepomis gibbosus*) [6]. Similarly, according to Saraiva et al. [7], the environmental enrichment was capable of inducing morphological differentiation through phenotypic plasticity, probably generating phenotypes more adapted to exploiting a complex environment.

*Procambarus clarkii,* commonly known as crayfish, belongs to the Decapoda, Cambaridae family. *Procambarus* is one of China’s major freshwater aquaculture crayfish. As of 2023, with a national crayfish aquaculture production of 2.8907 million tons, the total value of industrial output reached USD 63.31 billion [8]. In recent years, with the continuous expansion of aquaculture scale, excessive inbreeding has led to germplasm degradation, a high incidence of disease, individual miniaturization, and other problems that are becoming more and more prominent, seriously restricting the sustainable development of the crayfish industry, so it is imperative to carry out the selection of excellent traits of crayfish and the cultivation of good seed.

It is well known that for decapods such as shrimps and crabs, growth-related morphological traits are important references for selection and breeding. The Ecuadorian industry uses genetic parameters of growth and morphological traits of the Pacific South American white shrimp (Penaeus vannamei) as an important reference for selective breeding programs [9]. At the same time, morphological traits are also an important basis for revealing the evolutionary patterns [10] and systematic differentiation of organisms [11]. Therefore, the study of crayfish morphological traits is of scientific value for the systematic understanding of the life history evolution of crayfish as well as for the future selective breeding of crayfish.

Currently, studies on crayfish are mainly focused on environmental stress [12], culture diseases [13], nutritional quality [14], seed breeding [15], etc., and there are still few studies on morphology. Zhang et al. analyzed the effects of morphological traits on body weight of crayfish of different sexes by using path analysis [16]. However, the effects of morphological traits on the body weight of crayfish under different growing environments have not been reported. In the present study, the same batch of crayfish fry was cultured in the same cycle according to three modes of aquaculture: pond, paddy field, and aquaculture barrel. Using correlation analysis, pathway analysis, regression analysis, and gray relation analysis, we investigated the effects of different growing environments and sexes on the morphological traits of crayfish (*Procambarus clarkii*) and the correlation between traits and body weight in order to provide a scientific basis for the selection of the most suitable cultivation mode in the artificial breeding process for crayfish in the later stage of development.

### Definitions

D1-♀ are populations of female crayfish cultured in a pond; D1-♂ are populations of male crayfish cultured in a pond; D2-♀ are populations of female crayfish cultured in a paddy field; D2-♂ are populations of male crayfish cultured in a paddy field; D3-♀ are populations of female crayfish cultured in an aquaculture barrel; and D3-♂ are populations of male crayfish cultured in an aquaculture barrel.

## 2. Materials and Methods

### 2.1. Test Materials

In this study, juvenile crayfish (F1) hatched from the same population (F0) in East Dongting Lake (E: 113°01′36″, N: 29°38′26″, Xikou Fishing Village, Junshan District, Yueyang City 41400, Hunan Province, China) were cultured in different growing environments: pond (***D*1**), paddy field (***D*2**), and aquaculture barrel (***D*3**). The environmental parameters of each culture were as follows: ***D*1**: pond, area 6666.67 square meters, water depth 1.5 m, dissolved oxygen 5.8–6.8 mg/L, water transparency 25–35 cm, water temperature 22–28 °C, pH value 7.8–8.5, ammonia nitrogen < 0.5 mg/L, and planting aquatic plants (plant spacing 1 m × 1 m). ***D*2**: paddy field, area 6666.67 square meters, water depth 1.0–1.5 m, dissolved oxygen 5.0–5.8 mg/L, water transparency 30–40 cm, water temperature 22–30 °C, pH value 6.8–7.5, ammonia nitrogen < 0.5 mg/L, and planting aquatic plants (plant spacing 1 m × 1 m). ***D*3**: aquaculture barrel, area 113.04 square meters (three replicates), water depth 1.0–1.2 m, dissolved oxygen 5.8–6.8 mg/L, water transparency 30–40 cm, water temperature 22–28 °C, pH value 7.5–8.3, ammonia nitrogen < 0.5 mg/L, and placement bionic plants (plant spacing 1 m × 1 m). Crayfish were cultured at the same unit density (6–8 tails per square) for 60 days in each environment, fed the same diet (32% crude protein and 5% crude fat) at 5% of the total weight twice a day (6:00, 18:00). At the end of the culture period, we randomly selected female and male crayfishes, 50 individuals each, from each environment to measure their morphological traits.

### 2.2. Measurement of Morphometric Traits and Weight

**Figure 1:**
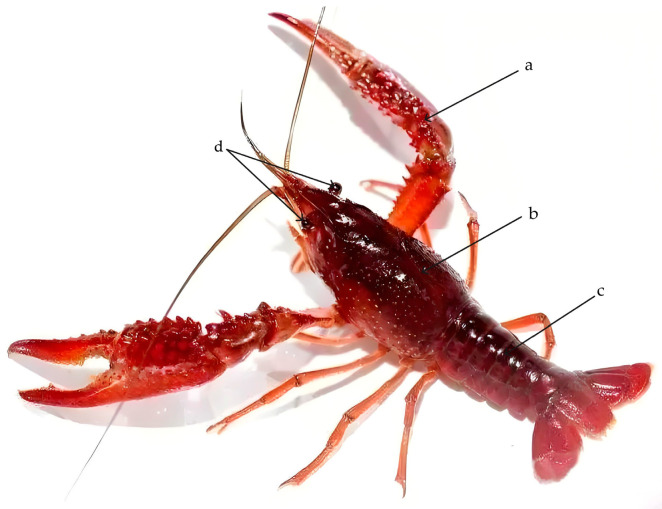
Names of the main measuring parts of the body of crayfish. a: chelicerae; b: cephalothorax; c: caudal peduncle; d: eyes.

The live crayfishes were transported back to the laboratory, for anesthesia treatment with clove oil; a test anesthetic with low irritation was applied at a concentration of 1:10,000 clove oil to water by volume, until the crayfish were quiet and stopped moving. Body surface moisture was cleaned with a dry towel, an electronic balance (precision to 0.0001 g) (*Y*), and a vernier caliper (precision length to 0.01 mm) were used for measuring 10 morphological traits. Body weight (*Y*) and 10 indicators of morphological traits, namely full length (*X*_1_), body length (*X*_2_), chelicerae length (*X*_3_), chelicerae weight (*X*_4_), cephalothorax length (*X*_5_), cephalothorax width (*X*_6_), cephalothorax height (*X*_7_), eye spacing (*X*_8_), caudal peduncle length (*X*_9_), and caudal peduncle weight (*X*_10_), were measured, respectively (Figure 1).

### 2.3. Data Analysis Method

#### 2.3.1. Correlation and Pathway Analysis

Correlation analysis could measure the degree of correlation between each trait and body weight, while pathway analysis could calculate the size of direct, indirect, and decision-making effects of each morphological trait on body weight (i.e., pass-through coefficient, indirect pass-through coefficient, and decision-making coefficient), which truly reflected the role of each trait in relation to body weight.

Microsoft Excel 2003 and SPSS software (version 19.0) were used to organize and count the measurement data for each morphological trait, conduct correlation analysis, pathway analysis, decision coefficient calculation, and establish multiple regression equations for morphological traits and body weight by using the multiple stepwise linear regression method with body weight as the dependent variable and other morphological traits as the independent variables [17].

#### 2.3.2. Gray Relation Analysis

Gray correlation analysis was not limited by the number of samples. The analysis process did not take into account the mutual influence of independent variables, and only analyzed the correlation coefficients between the respective variables and the dependent variable.

Based on the theory of the gray relation analysis system [18], the body weight and 10 morphological traits of crayfish in this measurement were considered a gray correlation system. The body weight was taken as the reference sequence, and the 10 morphological traits as the comparison sequence, and the data were dimensionless using standard deviation to calculate the gray correlation coefficients and correlation degrees of the 10 morphological traits. The calculation formulas were as follows:

$$x_{1}^{\prime }(k)=\frac{x_{i}\left(k\right)-{\bar{x}}_{i}}{S_{i}}$$

$$\delta i\left(k\right)=\frac{min\left|x_{0}^{\prime }\left(k\right)-x_{i}^{\prime }(k)\right|+\rho max\left|x_{0}^{\prime }\left(k\right)-x_{i}^{\prime }(k)\right|}{\left|x_{0}^{\prime }\left(k\right)-x_{i}^{\prime }(k)\right|+\rho max\left|x_{0}^{\prime }(k)-x_{i}^{\prime }(k)\right|}$$

$$r_{i}=\frac{1}{n}\sum_{k=1}^{n}\delta_{1}(k)$$

in which $x_{1}^{\prime }\left(k\right)$ is the data after dimensionless processing, $x_{1}^{\prime }\left(k\right)$ is the original measurement value of the phenotypic trait, $\bar{x_{i}}$ is the mean value of the phenotypic trait, and $S_{i}$ is the standard deviation of the phenotypic trait. $\delta i\left(k\right)$ is the gray correlation coefficient of morphological traits, ρ  is the discrimination coefficient, which is set in this paper as  ρ=0.5, $\;min\left|x_{0}^{\prime }\left(k\right)-x_{i}^{\prime }(k)\right|$ indicates the minimum value in the absolute difference of morphological traits, $max\left|x_{0}^{\prime }\left(k\right)-x_{i}^{\prime }(k)\right|\;$ indicates the maximum value in the absolute difference of morphological traits, $r_{i}$ indicates the correlation degree of morphological traits $\;x_{i}$ on body weight $\;{\;x}_{0}$, and finally, the correlation order is arranged according to the size of correlation degree, and the smaller the correlation order is, the more important it is for body weight.

## 3. Results

### 3.1. Statistical Analysis of Parameters and Correlation Coefficients of Crayfish under Different Cultivation Modes

The morphological traits of female and male crayfish in different cultivation modes (Table 1, Table 2 and Table 3) showed that the coefficient of variation for cheliped weight (*X*_4_) was the highest for both female and male crayfish in all cultivation modes, and greater for the male crayfish (37.76% to 66.74%) than the female crayfish (28.58% to 38.67%). In the ***D*1** and ***D*2** modes, the coefficient of variation for cheliped weight (*X*_4_) was higher than that for the raw crayfish cultivated in the ***D*3** mode, especially for the male crayfish. This indicates that chelate weight was the most unstable and susceptible trait among the 10 morphological traits measured in crayfish.

The correlations between male and female traits of crayfish in each cultivation mode:

In pond (***D*1**), female morphological traits were significantly associated with their body mass (*p* < 0.05). The top five traits were cephalothorax length (*X*_5_) (0.909) > cephalothorax width (*X*_6_) (0.906) > full length (*X*_1_) (0.885) > body length (*X*_2_) (0.884) > tail weight (*X*_10_) (0.867). Except for eye spacing (*X*_8_) in male crayfish, all the other traits showed a significant correlation with body mass (*p* < 0.05). The top five traits with the strongest correlation were chelicerae weight (*X*_4_) (0.913) > chelicerae length (*X*_3_) (0.902) > body length (*X*_2_) (0.887) > caudal peduncle weight (*X*_10_) (0.876) > cephalothorax width (*X*_6_) (0.832) (Table 4).

In paddy field (***D*2**), the morphological traits of female crayfish, except for eye spacing (*X*_8_), showed a significant correlation with body mass (*p* < 0.05). The top five traits with the strongest correlation were full length (*X*_1_) (0.960) > body length (*X*_2_) (0.944) > chelicerae weight (*X*_4_) (0.942) > caudal peduncle weight (*X*_10_) (0.921) > cephalothorax length (*X*_5_) (0.900). All traits and body mass in male crayfish had significant correlation levels (*p* < 0.05), and the top five traits with the strongest correlation were cephalothorax length (*X*_5_) (0.977) > chelicerae weight (*X*_4_) (0.976) > caudal peduncle weight (*X*_10_) (0.973) > full length (*X*_1_) (0.972) > body length (*X*_2_) (0.970) (Table 5).

In aquaculture barrel (***D*3**), the correlation coefficients between morphological traits and body weight of female and male crayfish were significant (*p* < 0.05). The top five traits of female crayfish with the strongest correlation were **full length** (*X*_1_) (0.938) > **chelicerae weight** (*X*_4_) (0.917) > **body length** (*X*_2_) (0.911) > **caudal peduncle weight** (*X*_10_) (0.910) > **chelicerae length** (*X*_3_) (0.872); and the top five traits of male crayfish with the strongest correlation were **full length** (*X*_1_) (0.940) > **chelicerae weight** (*X*_4_) (0.910) > **cephalothorax width** (*X*_6_) (0.881) > **caudal peduncle weight** (*X*_10_) (0.878) > **chelicerae length** (*X*_3_) (0.840) (Table 6).

### 3.2. Effect of Morphological Traits of Crayfish on Body Weight under Different Cultivation Modes

The influence coefficient of morphological traits on body weight consists of three parts: the pathway coefficient (direct effect), the indirect pathway coefficient (indirect effect), and the decision coefficient (decision effect). As can be seen from Table 7 and Table 8, under the pond cultivation mode (***D*1**), the four traits of chelicerae weight (*X*_4_), cephalothorax length (*X*_5_), cephalothorax height (*X*_7_), and caudal peduncle weight (*X*_10_) had significant direct effects on the body weight of female crayfish (*p* < 0.05). The effect sizes were *P*_4_*_y_* (0.412), *P*_5_*_y_* (0.259), *P*_7_*_y_* (0.200), and *P*_10_*_y_* (0.242), respectively (Table 7). The indirect effects of chelicerae weight (*X*_4_) on body weight (*Y*) through cephalothorax length (*X*_5_), cephalothorax height (*X*_7_), and caudal peduncle weight (*X*_10_) were 0.183, 0.101, and 0.154. The indirect effects of the cephalothorax length (*X*_5_) on the body weight (*Y*) through chelicerae weight (*X*_4_), cephalothorax height (*X*_7_), and caudal peduncle weight (*X*_10_) were 0.291, 0.143, and 0.215. The indirect effects of cephalothorax height (*X*_7_) on body weight (*Y*) through chelicerae weight (*X*_4_), cephalothorax length (*X*_5_), and caudal peduncle weight (*X*_10_) were 0.208, 0.186, and 0.160. And the indirect effects of caudal peduncle weight (*X*_10_) on body weight (*Y*) through chelicerae weight (*X*_4_), cephalothorax length (*X*_5_), and cephalothorax height (*X*_7_) were 0.262, 0.230, and 0.132, respectively. The order of the decision coefficients was as follows: *X*_4_ (0.531) > *X*_5_ (0.404) > *X*_10_ (0.361) > *X*_7_ (0.262) > 0, indicating that chelicerae weight (*X*_4_), cephalothorax length (*X*_5_), cephalothorax height (*X*_7_), and caudal peduncle weight (*X*_10_) of female crayfish in the pond cultivation mode (***D*1**) could all affect their body weight (*Y*). For male crayfish, body length (*X*_2_), chelicerae weight (*X*_4_), and cephalothorax width (*X*_6_) had significant direct effects on body weight (*Y*) (*p* < 0.05), and the effect sizes were *P*_2_*_y_* (0.262), *P*_4_*_y_* (0.501), and *P*_6_*_y_* (0.286), respectively (Table 7). The indirect effects of body length (*X*_2_) on body weight (*Y*) through chelicerae weight (*X*_4_) and cephalothorax width (*X*_6_) were 0.408 and 0.217. The indirect effects of chelicerae weight (*X*_4_) on body weight (*Y*) through body length (*X*_2_) and cephalothorax width (*X*_6_) were 0.213 and 0.198. The indirect effects of cephalothorax width (*X*_6_) on body weight (*Y*) through body length (*X*_2_) and chelicerae weight (*X*_4_) were 0.199 and 0.347. The order of decision coefficients was *X*_4_ (0.664) > *X*_6_ (0.394) > *X*_2_ (0.391) > 0, indicating that the body length (*X*_2_), chelicerae weight (*X*_4_), and cephalothorax width (*X*_6_) played an important role in enhancing the body weight (*Y*) of male crayfish in the pond mode (Table 8).

In the paddy field mode (***D*2**), the direct effects of full length (*X*_1_), chelicerae weight (*X*_4_), cephalothorax length (*X*_5_), and caudal peduncle weight (*X*_10_) on the body weight (*Y*) of female crayfish were significant (*p* < 0.05). The effect sizes were *P*_1_*_y_* (0.231), *P*_4_*_y_* (0.350), *P*_5_*_y_* (0.222), and *P*_10_*_y_* (0.246) (Table 9). The indirect effects of full length (*X*_1_) on body weight (*Y*) through chelicerae weight (*X*_4_), cephalothorax length (*X*_5_), and caudal peduncle weight (*X*_10_) were 0.325, 0.196, and 0.209, respectively. And the indirect effects of chelicerae weight (*X*_4_) on body weight (*Y*) through full length (*X*_1_), cephalothorax length (*X*_5_), and caudal peduncle weight (*X*_10_) were 0.214, 0.171, and 0.207. The indirect effects of cephalothorax length (*X*_5_) on body weight (*Y*) through full length (*X*_1_), chelicerae weight (*X*_4_), and caudal peduncle weight (*X*_10_) were 0.204, 0.270, and 0.205. The indirect effects of caudal peduncle weight (*X*_10_) on body weight (*Y*) through full length (*X*_1_), chelicerae weight (*X*_4_), and cephalothorax length (*X*_5_) were 0.196, 0.295, and 0.185, respectively. The order according to the size of the decision coefficient was *X*_4_ (0.537) > *X*_10_ (0.393) > *X*_1_ (0.390) > *X*_5_ (0.350) > 0, indicating that the full length (*X*_1_), chelicerae weight (*X*_4_), cephalothorax length (*X*_5_), and caudal peduncle weight (*X*_10_) of female crayfish under the paddy-field mode could promote their body weight (*Y*) (Table 10). However, the direct effects of chelicerae weight (*X*_4_) and cephalothorax length (*X*_5_) on the body weight of male crayfish were significant (*p* < 0.05), and the effect sizes were *P*_4_*_y_* (0.517) and *P*_5*y*_ (0.479) (Table 9). The indirect effect of chelicerae weight (*X*_4_) on body weight (*Y*) through cephalothorax length (*X*_5_) was 0.460, and the indirect effect of cephalothorax length (*X*_5_) on body weight (*Y*) through chelicerae weight (*X*_4_) was 0.496. The order of decision coefficients was *X*_4_ (0.743) > *X*_5_ (0.706) > 0, indicating that chelicerae weight (*X*_4_) and cephalothorax length (*X*_5_) had an increasing effect on the body weight (*Y*) of male crayfish under the paddy-field mode (Table 10).

As can be seen from Table 11 and Table 12, in the aquaculture barrel mode (***D*3**), the direct effect of only chelicerae length (*X*_3_) and caudal peduncle weight (*X*_10_) on body weight (*Y*) of female crayfish was significant (*p* < 0.05), and the effect sizes were *P*_3_*_y_* (0.495) and *P*_10_*_y_* (0.597), respectively (Table 11). The indirect effect of chelicerae length (*X*_3_) on body weight (*Y*) through caudal peduncle weight (*X*_10_) was 0.377, while the indirect effect of caudal peduncle weight (*X*_10_) on body weight (*Y*) through chelicerae length (*X*_3_) was 0.313. The order of the decision coefficient was *X*_10_ (0.730) > *X*_3_ (0.618) > 0, indicating that only chelicerae length (*X*_3_) and caudal peduncle weight (*X*_10_) could improve the body weight (*Y*) of female crayfish in barrel mode (Table 12). The direct effect of full length (*X*_1_) and chelicerae weight (*X*_4_) on body weight (*Y*) of male crayfish was significant (*p* < 0.05), and the effect sizes were *P*_1_*_y_* (0.611) and *P*_4_*_y_* (0.378) (Table 11). The indirect effect of full length (*X*_1_) on body weight (*Y*) through chelicerae weight (*X*_4_) was 0.532, while the indirect effect of chelicerae weight (*X*_4_) on body weight (*Y*) through full length (*X*_1_) was 0.329. The order of the decision coefficient was *X*_1_ (0.775) > *X*_4_ (0.545) > 0, indicating that the full length (*X*_1_) and chelate foot weight (*X*_4_) of male crayfish could improve their body weight (*Y*) (Table 12).

### 3.3. Multiple Regression Equations for Morphological Traits and Body Mass of Crayfish under Different Cultivation Modes

According to the principle of partial regression coefficient, the test of regression constant significance, and the complex correlation analysis on morphological traits and body weight (Table 13, Table 14 and Table 15), with body weight (*Y*) as the dependent variable and morphological trait (*Xi*) as the independent variable, the regression equations for females and males in each cultivation mode were constructed as follows:

*Y*_D1-♀_ = −29.803 + 1.249*X*_4_ + 0.505*X*_5_ + 0.701*X*_7_ + 1.483*X*_10_; *Y*_D1-♂_ = −40.881 + 0.39*X*_2_ + 0.845*X*_4_ + 1.142*X*_6_

*Y*_D2-♀_ = −12.248 + 0.088*X*_1_ + 1.098*X*_4_ + 0.275*X*_5_ + 0.904*X*_10_; *Y*_D2-♂_= −24.871 + 1.177*X*_4_ + 0.902*X*_5_

*Y*_D3-♀_ = −22.476 + 0.432*X*_3_ + 3.153*X*_10_; *Y*_D3-♂_ = −34.434 + 0.363*X*_1_ + 0.669*X*_4_

where *Y*_D1-♀_, *Y*_D1-♂_, *Y*_D2-♀_, *Y*_D2-♂_, *Y*_D3-♀_, *Y*_D3-♂_ were the body weights of pond female crayfish (D1-♀), pond male crayfish (D1-♂), paddy-field female crayfish (D2-♀), paddy-field male crayfish (D2-♂), aquaculture barrel female crayfish (D3-♀), and aquaculture barrel male crayfish (D3-♂), respectively. *X*_1_, *X*_2_, *X*_3_, *X*_4_, *X*_5_, *X*_6_, *X*_7_, and *X*_10_ represent full length, body length, chelicerae length, chelicerae weight, cephalothorax length, cephalothorax width, cephalothorax height, and caudal peduncle weight, respectively.

### 3.4. Gray Correlation Analysis of Morphological Traits and Body Weight of Crayfish under Different Cultivation Modes

The results of the gray correlation analysis of morphological traits and body weight of crayfish under different cultivation modes are shown in Table 16, Table 17 and Table 18. The correlation coefficients for female and male crayfish cultured in pond (***D*1**) were 0.744~0.855 and 0.756~0.866, respectively. The correlations between body weight and morphological traits of female crayfish were as follows: caudal peduncle weight (*X*_10_) > chelicerae length (*X*_3_) > full length (*X*_1_) > cephalothorax width (*X*_6_) > cephalothorax height (*X*_7_) > body length *(X_2_*) > cephalothorax length (*X*_5_) > caudal peduncle length (*X*_9_) > chelicera length (*X*_4_) > eye spacing (*X*_8_). And the correlation order between body weight and morphological traits in male crayfish was caudal peduncle weight (*X*_10_) > chelicerae length (*X*_3_) > cephalothorax height (*X*_7_) > cephalothorax width (*X*_6_) > caudal peduncle length (*X*_9_) > body length (*X*_2_) > full length (*X*_1_) > cephalothorax length (*X*_5_) > eye spacing (*X*_8_) > chelicerae weight (*X*_4_) (Table 16).

The correlation coefficients of female and male crayfish cultured in paddy field mode (***D*2**) were 0.717~0.857 and 0.686~0.877, respectively. The correlations between body weight and morphological traits of female crayfish were as follows: chelicerae length (*X*_3_) > caudal peduncle weight (*X*_10_) > full length (*X*_1_) > cephalothorax height (*X*_7_) > cephalothorax length (*X*_5_) > cephalothorax width (*X*_6_) > body length (*X*_2_) > caudal peduncle length (*X*_9_) > chelicerae weight (*X*_4_) > eye spacing (*X*_8_). And the correlation order between body weight and morphological traits in male crayfish was caudal peduncle weight (*X*_10_) > chelicerae length (*X*_3_) > full length (*X*_1_) > cephalothorax width (*X*_6_) > cephalothorax height (*X*_7_) > cephalothorax length (*X*_5_) > chelicerae weight (*X*_4_) > eye spacing (*X*_8_) > body length (*X*_2_) > caudal peduncle length (*X*_9_) (Table 17).

The correlation coefficients of female and male crayfish cultured in aquaculture barrel (***D*3**) were 0.718~0.801 and 0.632~0.750, respectively. The correlations between body weight and morphological traits of female crayfish were as follows: caudal peduncle weight (*X*_10_) > cephalothorax width (*X*_6_) > full length (*X*_1_) > chelicerae weight (*X*_4_) > body length (*X*_2_) > cephalothorax length (*X*_5_) > chelicerae length (*X*_3_) > cephalothorax height (*X*_7_) > caudal peduncle length (*X*_9_) > eye spacing (*X*_8_). And the correlation order between body weight and morphological traits in male crayfish was caudal peduncle weight (*X*_10_) > chelicerae weight (*X*_4_) > full length (*X*_1_) > chelicerae length (*X*_3_) > caudal peduncle length (*X*_9_) > cephalothorax width (*X*_6_) > cephalothorax height (*X*_7_) > cephalothorax length (*X*_5_) > body length (*X*_2_) > eye spacing (*X*_8_) (Table 18).

## 4. Discussion

### 4.1. Differences in Morphological Traits and Correlation with Body Weight of Crayfish under Different Cultivation Modes

It is well known that the morphological traits of animals are influenced by both genetics and environment, especially for aquatic animals such as crayfishes and crabs, which grow out of their shells, and their morphological traits are easily affected by parameters of the environment in which they live, such as light [19], water temperature [20], bait [19], concealment [19], etc. In this study, we purposely chose the same population with the same batch of breeding offspring of crayfish for simultaneous cultivation in three different culture environments, namely, pond (***D*1**), paddy field (***D*2**), and aquaculture barrel (***D*3**), to study the effects of different culture modes (environments) on their morphological traits. It was found that the coefficient of variation of chelicerae weight (*X*_4_) of crayfish under each culture mode was the largest, and the coefficient of variation of chelicerae weight of males (28.58%~38.67%) was larger than that of females (37.76%~66.74%), and the coefficients of variation of chelicerae weight (*X*_4_) of female and male crayfish under the culture modes of ***D*1** and ***D*2** were larger than those of the crayfish cultured in ***D*3**, especially that of males, which showed the most obvious performance.

The chelicerae are the main organs that the crayfish and crab use to survive competition and resist invasion, and the paddy-culture environment is more unfavorable than the pond and aquaculture barrel environment in terms of water depth, concealment, bait abundance, etc. Therefore, the coefficient of variation of the chelicerae of crayfish cultured in paddy fields was the largest (38.67% in females and 66.74% in males), which indicates that the cheliped size of crayfish is the trait most susceptible to the influence of the culture environment, and it also indicates that the males are more adept at fighting.

A correlation analysis was conducted between the measured morphological traits and body weight of crayfish cultivated in each culture mode (environment) to measure the closeness of the relationship with body weight. The results showed that all morphological traits except for eye spacing (*X*_8_) were significantly correlated with body weight (*p* < 0.05) and could be used as an indirect basis for selecting the body weight of crayfish in the artificial selection process. However, it was also found that the correlation coefficients between morphological traits and body weight of crayfish of different sexes in the same mode and of the same sex in different modes were not the same, indicating that different modes and sexes would lead to differences in morphological traits of crayfish. This is similar to the findings of Zhu et al. [21] and Zhang et al. [16].

### 4.2. Effects of Morphological Traits on Body Weight and Identification of Key Traits in Crayfish under Different Culture Modes

Correlation analysis can only measure the degree of relationship between morphological traits and body weight and cannot clarify the specific scale of their role or degree of influence on body weight. Pathway analysis can calculate the direct, indirect, and decision-making effects of each morphological trait on body weight (i.e., passage coefficient, indirect passage coefficient, and decision-making coefficient), thus accurately reflecting the role of each trait in relation to body weight, and this method has been widely used in the genetic selection of fish [22,23,24]. The results of the pathway analysis in this study showed that the direct and indirect effects and decision coefficients of chelicerae weight (*X*_4_) of female and male crayfish on body weight were greater than those of other traits of crayfish cultured in both pond (***D*1**) and paddy field (***D*2**) modes, and this was the key trait influencing the body weight of crayfish under these two culture modes. In the aquaculture barrel mode (***D*3**), the direct and indirect effects of caudal peduncle weight (*X*_10_) were greater than those of other traits, and this was the key trait affecting body weight in females, whereas in males, full length (*X*_1_) was the key trait affecting body weight. This finding further confirms the significant influence of culture mode (environment) on the morphological traits of crayfish. Compared with captive culture in aquaculture barrel mode (***D*3**), crayfish in pond (***D*1**) and paddy field (***D*2**) modes had more competitive pressure for survival, such as food grabbing and enemy protection, so the preferential development of robust chelipeds was more consistent with the adaptation of their survival to the environment [16]. In addition, although female and male crayfish in the same aquaculture barrel (***D*3**) did not have competitive pressure such as food grabbing, there were obvious sex differences in individual morphological development, which involved preparing for subsequent mating and breeding [25,26]. Although traits such as chelicerae weight (*X*_4_), caudal peduncle weight (*X*_10_), and full length (*X*_1_) were the key traits affecting the body weight in each mode, their decision coefficients were less than 0.85, which indicated that there were other key traits affecting the body weight of both female and male crayfish in each mode. The total decision coefficient (*R*^2^) for the female crayfish cultured in pond (***D*1**) was 0.947 (>0.85) with the introduction of trait variables such as cephalothorax length (*X*_5_), cephalothorax height (*X*_7_), and caudal peduncle weight (*X*_10_). The result suggested that 94.7% of the variance in body weight of the D1-♀ group originated from these key traits, so the following regression equation was constructed: *Y*_D1-__♀_ = −29.803 + 1.249*X*_4_ + 0.505*X*_5_ + 0.701*X*_7_ + 1.483*X*_10_ (*R*^2^ = 0.947); similarly, the regression equations for female and male crayfish in the other modes were as follows: *Y*_D1-__♂_ = −40.881 + 0.39*X*_2_ + 0.845*X*_4_ + 1.142*X*_6_ (*R*^2^ = 0.927); *Y*_D2-__♀_ = −12.248 + 0.088*X*_1_ + 1.098*X*_4_ + 0.275*X*_5_ + 0.904*X*_10_ (*R*^2^ = 0.977); *Y*_D2-__♂_ = −24.871 + 1.177*X*_4_ + 0.902*X*_5_ (*R*^2^ = 0.973); *Y*_D3-__♀_ = −22.476 + 0.432*X*_3_ + 3.153*X*_10_ (*R*^2^ = 0.976); and*Y*_D3-__♂_ = −34.434 + 0.363*X*_1_ + 0.669*X*_4_ (*R*^2^ = 0.918).

Gray correlation analysis was performed to calculate the gray correlation coefficients and correlations between each morphological trait (independent variable) and body weight (dependent variable) by dimensionless quantification of the measured data of 10 morphological traits using standard deviation. However, the gray correlation analysis is not limited by the number of samples, so the results obtained by cross-comparison of the two analysis methods are more accurate.

## 5. Conclusions

By comparing the morphological traits retained by the pathway analysis of female and male crayfish populations cultivated under each culture mode (environment) with the top five traits ranked by the gray correlation analysis in terms of correlation, it was concluded that the traits most correlated with body weight of female crayfish cultured in ponds (D1-♀) were caudal peduncle weight (correlation ranked 1) and cephalothorax height (correlation ranked 5); in pond male crayfish populations (D1-♂), the trait most correlated with body weight was cephalothorax width. The most relevant traits for body weight in the group of female crayfish cultured in paddy fields (D2-♀) were caudal peduncle weight (correlation rank 2), full length (correlation rank 3), and cephalothorax length (correlation rank 5); whereas the group of male crayfish cultured in paddy field mode (D2-♂), the most relevant trait for body weight was cephalothorax length (correlation rank 5). The most relevant trait for body weight in the group of female crayfish farmed in aquaculture barrels (D3-♀) was caudal peduncle weight (correlation rank 1); for the group of male crayfish cultured in aquaculture barrels (D3-♂), the most relevant traits for body weight were chelicerae weight (correlation rank 2) and full length (correlation rank 3). The results of the two analyses were in good agreement, and the conclusions were reliable.

Overall, in terms of individual weight gain, female crayfish were more suitable for the pond culture model, obtaining the greatest weight of the caudal peduncle. And in terms of individual resistance, male crayfish were more suitable for the aquaculture barrel model, obtaining the most robust chelicerae (greatest weight and length of the chelicerae).

## Figures and Tables

**Table 1:** Statistical analysis of measurement data of male and female crayfishes cultured in pond.

Group	Sex	Item	*Y*/g	*X*_1_/mm	*X*_2_/mm	*X*_3_/mm	*X*_4_/g	*X*_5_/mm	*X*_6_/mm	*X*_7_/mm	*X*_8_/mm	*X*_9_/mm	*X*_10_/g
***D*1**	♀	Mean	33.03	157.14	107.11	75.78	6.17	52.92	23.99	25.34	16.02	54.53	7.18
		Standard deviation	6.71	12.33	6.95	8.12	2.21	3.44	1.68	1.92	1.39	9.52	1.09
		Skewness	1.35	0.51	0.25	0.78	1.49	0.81	0.55	0.80	−0.69	−4.21	0.81
		Kurtosis	3.42	0.54	0.82	0.55	3.17	1.37	0.20	0.04	1.27	22.15	0.34
		CV/%	20.32	7.84	6.49	10.72	35.87	6.51	7.00	7.57	8.68	17.45	15.24
	♂	Mean	32.45	157.81	101.53	87.18	8.82	51.20	23.05	25.35	14.87	52.35	6.00
		Standard deviation	7.88	16.49	5.29	10.60	4.68	3.49	1.97	3.04	1.20	3.72	1.12
		Skewness	1.44	0.23	0.83	0.89	1.79	0.00	1.05	0.71	−1.10	0.11	0.60
		Kurtosis	2.43	2.55	0.47	1.03	3.50	0.71	0.64	0.17	2.75	0.11	0.53
		CV/%	24.28	10.45	5.21	12.16	53.02	6.81	8.56	12.00	8.05	7.11	18.66

**Table 2:** Statistical analysis of measurement data of male and female crayfishes cultured in paddy field.

Group	Sex	Item	*Y*/g	*X*_1_/mm	*X*_2_/mm	*X*_3_/mm	*X*_4_/g	*X*_5_/mm	*X*_6_/mm	*X*_7_/mm	*X*_8_/mm	*X*_9_/mm	*X*_10_/g
***D*2**	♀	Mean	19.14	130.77	92.01	62.07	3.19	44.74	19.86	21.37	14.10	47.49	4.51
		Standard deviation	3.87	10.15	5.90	9.54	1.23	3.13	1.53	1.61	1.61	2.85	1.05
		Skewness	1.84	1.42	2.16	2.14	1.98	1.41	1.16	0.02	−1.54	1.82	2.81
		Kurtosis	5.57	3.40	6.81	6.04	6.35	3.04	3.28	1.48	2.33	5.34	10.96
		CV/%	20.24	7.76	6.41	15.37	38.67	6.99	7.72	7.52	11.43	5.99	23.36
	♂	Mean	19.44	135.67	87.31	69.83	4.89	42.74	18.94	19.79	13.29	45.82	3.84
		Standard deviation	8.01	18.14	8.23	12.74	3.26	4.59	2.64	2.39	1.54	4.31	1.41
		Skewness	1.43	0.75	0.99	0.75	1.13	0.88	1.15	1.42	0.06	1.08	1.16
		Kurtosis	2.15	−0.12	0.91	−0.50	0.60	0.49	0.67	1.23	−0.35	0.89	0.59
		CV/%	41.19	13.37	9.43	18.24	66.74	10.74	13.92	12.09	11.57	9.40	36.57

**Table 3:** Statistical analysis of measurement data of male and female crayfishes cultured in aquaculture barrel.

Group	Sex	Item	*Y*/g	*X*_1_/mm	*X*_2_/mm	*X*_3_/mm	*X*_4_/g	*X*_5_/mm	*X*_6_/mm	*X*_7_/mm	*X*_8_/mm	*X*_9_/mm	*X*_10_/g
***D*3**	♀	Mean	28.69	150.37	101.05	73.29	5.89	49.13	22.28	23.56	15.08	53.38	6.18
		Standard deviation	5.00	9.06	4.63	5.73	1.68	2.12	1.40	1.66	1.86	2.78	0.95
		Skewness	0.77	0.32	0.34	0.37	1.28	0.64	−0.37	0.51	−2.03	−0.19	0.50
		Kurtosis	0.01	−0.56	−0.75	−0.02	1.89	−0.30	0.76	0.45	6.54	−1.11	−0.56
		CV/%	17.42	6.02	4.58	7.82	28.58	4.32	6.29	7.04	12.33	5.21	15.31
	♂	Mean	33.44	164.26	98.72	92.45	12.24	48.90	22.43	24.06	14.88	52.11	5.56
		Standard deviation	8.18	13.77	5.34	9.72	4.62	2.69	1.45	1.75	0.75	3.15	1.15
		Skewness	0.10	0.22	−0.52	−0.39	0.09	−0.48	0.48	−0.02	0.48	−0.18	0.40
		Kurtosis	−0.95	−0.95	−0.44	−1.05	−0.79	−0.68	−0.12	0.38	−0.76	−0.51	−1.18
		CV/%	24.47	8.38	5.41	10.51	37.76	5.51	6.47	7.26	5.01	6.04	20.60

**Table 4:** Correlation coefficients among characters of crayfish cultured in pond.

Group	Sex	Item	*Y*	*X*_1_	*X*_2_	*X*_3_	*X*_4_	*X*_5_	*X*_6_	*X*_7_	*X*_8_	*X*_9_	*X*_10_
***D*1**	♀	*Y*	1	0.885 **	0.884 **	0.797 **	0.851 **	0.909 **	0.906 **	0.754 **	0.400 *	0.473 **	0.867 **
		*X*_1_		1	0.829 **	0.931 **	0.871 **	0.814 **	0.826 **	0.621 **	0.406 *	0.434 **	0.726 **
		*X*_2_			1	0.666 **	0.691 **	0.947 **	0.895 **	0.700 **	0.505 **	0.422 *	0.877 **
		*X*_3_				1	0.890 **	0.676 **	0.713 **	0.558 **	0.330	0.339 *	0.577 **
		*X*_4_					1	0.706 **	0.729 **	0.506 **	0.301	0.391 *	0.637 **
		*X*_5_						1	0.892 **	0.717 **	0.499 **	0.366 *	0.889 **
		*X*_6_							1	0.736 **	0.440 **	0.447 **	0.868 **
		*X*_7_								1	0.462 **	0.477 **	0.660 **
		*X*_8_									1	0.120	0.503 **
		*X*_9_										1	0.448 **
		*X*_10_											1
	♂	*Y*	1	0.633 **	0.887 **	0.902 **	0.913 **	0.714 **	0.832 **	0.819 **	0.004	0.753 **	0.876 **
		*X*_1_		1	0.592 **	0.548 **	0.637 **	0.610 **	0.677 **	0.605 **	−0.062	0.355	0.607 **
		*X*_2_			1	0.813 **	0.814 **	0.803 **	0.758 **	0.793 **	0.046	0.738 **	0.805 **
		*X*_3_				1	0.939 **	0.611 **	0.650 **	0.641 **	0.201	0.726 **	0.794 **
		*X*_4_					1	0.619 **	0.693 **	0.722 **	0.086	0.707 **	0.813 **
		*X*_5_						1	0.714 **	0.629 **	0.137	0.322	0.691 **
		*X*_6_							1	0.901 **	−0.307	0.560 **	0.820 **
		*X*_7_								1	−0.275	0.658 **	0.799 **
		*X*_8_									1	−0.106	0.009
		*X*_9_										1	0.528 **
		*X*_10_											1

Note: * means significant difference (*p* < 0.05), ** indicates very significant difference (*p* < 0.01).

**Table 5:** Correlation coefficients among characters of crayfish cultured in paddy field.

Group	Sex	Item	*Y*	*X*_1_	*X*_2_	*X*_3_	*X*_4_	*X*_5_	*X*_6_	*X*_7_	*X*_8_	*X*_9_	*X*_10_
***D*2**	♀	*Y*	1	0.960 **	0.944 **	0.737 **	0.942 **	0.900 **	0.858 **	0.782 **	0.103	0.858 **	0.921 **
		*X*_1_		1	0.925 **	0.748 **	0.928 **	0.882 **	0.897 **	0.746 **	0.099	0.854 **	0.850 **
		*X*_2_			1	0.676 **	0.842 **	0.958 **	0.837 **	0.725 **	0.105	0.870 **	0.903 **
		*X*_3_				1	0.663 **	0.714 **	0.775 **	0.667 **	0.257	0.665 **	0.643 **
		*X*_4_					1	0.772 **	0.845 **	0.731 **	0.127	0.837 **	0.843 **
		*X*_5_						1	0.830 **	0.736 **	0.044	0.799 **	0.832 **
		*X*_6_							1	0.669 **	0.13	0.830 **	0.742 **
		*X*_7_								1	0.011	0.536 **	0.725 **
		*X*_8_									1	0.265	0.162
		*X*_9_										1	0.794 **
		*X*_10_											1
	♂	*Y*	1	0.972 **	0.970 **	0.948 **	0.976 **	0.977 **	0.963 **	0.946 **	0.783 **	0.930 **	0.973 **
		*X*_1_		1	0.976 **	0.984 **	0.974 **	0.984 **	0.961 **	0.949 **	0.824 **	0.936 **	0.974 **
		*X*_2_			1	0.937 **	0.941 **	0.992 **	0.975 **	0.951 **	0.845 **	0.956 **	0.981 **
		*X*_3_				1	0.970 **	0.954 **	0.941 **	0.925 **	0.788 **	0.909 **	0.948 **
		*X*_4_					1	0.960 **	0.941 **	0.950 **	0.775 **	0.886 **	0.957 **
		*X*_5_						1	0.975 **	0.949 **	0.838 **	0.947 **	0.977 **
		*X*_6_							1	0.956 **	0.825 **	0.944 **	0.982 **
		*X*_7_								1	0.820 **	0.927 **	0.964 **
		*X*_8_									1	0.796 **	0.850 **
		*X*_9_										1	0.946 **
		*X*_10_											1

Note: ** indicates very significant difference (*p* < 0.01).

**Table 6:** Correlation coefficients among characters of crayfish cultured in aquaculture barrel.

Group	Sex	Item	*Y*	*X*_1_	*X*_2_	*X*_3_	*X*_4_	*X*_5_	*X*_6_	*X*_7_	*X*_8_	*X*_9_	*X*_10_
***D*3**	♀	*Y*	1	0.938 **	0.911 **	0.872 **	0.917 **	0.838 **	0.774 **	0.721 **	0.586 **	0.755 **	0.910 **
		*X*_1_		1	0.862 **	0.940 **	0.863 **	0.797 **	0.775 **	0.656 **	0.568 **	0.721 **	0.782 **
		*X*_2_			1	0.744 **	0.744 **	0.886 **	0.755 **	0.761 **	0.556 **	0.816 **	0.878 **
		*X*_3_				1	0.841 **	0.733 **	0.715 **	0.651 **	0.500 *	0.588 **	0.632 **
		*X*_4_					1	0.764 **	0.713 **	0.535 *	0.578 **	0.581 **	0.799 **
		*X*_5_						1	0.628 **	0.788 **	0.405	0.651 **	0.790 **
		*X*_6_							1	0.592 **	0.532 *	0.639 **	0.671 **
		*X*_7_								1	0.324	0.499 *	0.714 **
		*X*_8_									1	0.245	0.556 **
		*X*_9_										1	0.735 **
		*X*_10_											1
	♂	*Y*	1	0.940 **	0.836 **	0.840 **	0.910 **	0.809 **	0.881 **	0.824 **	0.696 **	0.742 **	0.878 **
		*X*_1_		1	0.886 **	0.858 **	0.870 **	0.876 **	0.894 **	0.784 **	0.790 **	0.802 **	0.861 **
		*X*_2_			1	0.761 **	0.743 **	0.956 **	0.816 **	0.682 **	0.669 **	0.884 **	0.826 **
		*X*_3_				1	0.904 **	0.732 **	0.828 **	0.790 **	0.618 **	0.607 **	0.670 **
		*X*_4_					1	0.723 **	0.856 **	0.779 **	0.527 *	0.639 **	0.763 **
		*X*_5_						1	0.736 **	0.676 **	0.675 **	0.870 **	0.818 **
		*X*_6_							1	0.821 **	0.694 **	0.691 **	0.854 **
		*X*_7_								1	0.558 *	0.499 *	0.748 **
		*X*_8_									1	0.669 **	0.662 **
		*X*_9_										1	0.814 **
		*X*_10_											1

Note: * means significant difference (*p* < 0.05), ** indicates very significant difference (*p* < 0.01).

**Table 7:** Significance tests of partial regression coefficient and regression constant of morphological traits of crayfish cultured in pond.

Group	Sex	Model		Unstandardized Coefficients		Standardized Coefficients	T Value	Significant
				Regression Coefficients	Standard Error			
***D*1**	♀	1	Constant	−60.664	7.506		−8.082	0
			*X*_5_	1.771	0.142	0.909	12.509	0
		2	Constant	−38.11	6.71		−5.68	0
			*X*_5_	1.197	0.144	0.614	8.331	0
			*X*_4_	1.263	0.224	0.417	5.65	0
		3	Constant	−41.228	6.381		−6.642	0
			*X*_5_	0.901	0.21	0.394	3.651	0
			*X*_4_	1.263	0.216	0.359	5.04	0
			*X*_7_	0.741	0.446	0.292	2.619	0.003
		4	Constant	−29.803	7.00		−4.257	0
			*X*_5_	0.505	0.208	0.259	2.423	0.022
			*X*_4_	1.249	0.180	0.412	6.928	0
			*X*_7_	0.701	0.212	0.200	3.311	0.002
			*X*_10_	1.483	0.565	0.242	2.626	0.013
	♂	1	Constant	18.881	1.494		12.638	0
			*X*_4_	1.538	0.15	0.913	10.231	0
		2	Constant	−12.473	7.716		−1.616	0.122
			*X*_4_	1.09	0.157	0.647	6.927	0
			*X*_6_	1.532	0.373	0.384	4.107	0.001
		3	Constant	−40.881	14.844		−2.754	0.013
			*X*_4_	0.845	0.183	0.501	4.615	0
			*X*_6_	1.142	0.386	0.286	2.956	0.008
			*X*_2_	0.39	0.179	0.262	2.178	0.042

Note: Dependent variable: body weight.

**Table 8:** Effects of morphological traits on body weight of crayfish cultured in pond.

Group	Sex	Trait	Correlation Coefficients	Direct Effect	Indirect Effect					Decision Coefficients
					*X*_4_	*X*_5_	*X*_7_	*X*_10_	Total	
***D*1**	♀	*X*_4_	0.851	0.412	—	0.183	0.101	0.154	0.438	0.531
		*X*_5_	0.909	0.259	0.291	—	0.143	0.215	0.649	0.404
		*X*_7_	0.754	0.200	0.208	0.186	—	0.160	0.554	0.262
		*X*_10_	0.867	0.242	0.262	0.230	0.132	—	0.625	0.361
	♂				*X*_2_	*X*_4_	*X*_6_		Total	
		*X*_2_	0.887	0.262	—	0.408	0.217		0.625	0.391
		*X*_4_	0.913	0.501	0.213	—	0.198		0.411	0.664
		*X*_6_	0.832	0.286	0.199	0.347	—		0.546	0.394

**Table 9:** Significance tests of partial regression coefficient and regression constant of morphological traits of crayfish cultured in paddy field.

Group	Sex	Model		Unstandardized Coefficients		Standardized Coefficients	T Value	Significant
				Regression Coefficients	Standard Error			
***D*2**	♀	1	Constant	−28.770	2.993		−9.613	0
			*X*_1_	0.366	0.023	0.960	16.055	0
		2	Constant	−18.954	2.997		−6.325	0
			*X*_1_	0.243	0.031	0.637	7.849	0
			*X*_10_	1.397	0.298	0.380	4.684	0
		3	Constant	−11.439	4.419		−2.589	0.018
			*X*_1_	0.173	0.043	0.454	4.033	0.001
			*X*_10_	1.223	0.286	0.333	4.277	0
			*X*_4_	0.755	0.347	0.240	2.178	0.042
		4	Constant	−12.248	3.904		−3.138	0.005
			*X*_1_	0.088	0.050	0.231	1.760	0.005
			*X*_10_	0.904	0.280	0.246	3.231	0.004
			*X*_4_	1.098	0.332	0.350	3.302	0.004
			*X*_5_	0.275	0.105	0.222	2.607	0.017
	♂	1	Constant	−53.425	3.663		−14.585	0
			*X*_5_	1.705	0.085	0.977	20.002	0
		2	Constant	−24.871	8.728		−2.850	0.011
			*X*_5_	0.902	0.241	0.517	3.743	0.001
			*X*_4_	1.177	0.339	0.479	3.470	0.003

Note: Dependent variable: body weight.

**Table 10:** Effects of morphological traits on body weight of crayfish cultured in paddy field.

Group	Sex	Trait	Correlation Coefficients	Direct Effect	Indirect Effect					Decision Coefficients
					*X*_1_	*X*_4_	*X*_5_	*X*_10_	Total	
***D*2**	♀	*X*_1_	0.960	0.231	—	0.325	0.196	0.209	0.730	0.390
		*X*_4_	0.942	0.350	0.214	—	0.171	0.207	0.593	0.537
		*X*_5_	0.900	0.222	0.204	0.270	—	0.205	0.679	0.350
		*X*_10_	0.921	0.246	0.196	0.295	0.185	—	0.676	0.393
	♂				*X*_4_		*X*_5_		Total	
		*X*_4_	0.977	0.517	—		0.460		0.460	0.743
		*X*_5_	0.976	0.479	0.496		—		0.496	0.706

**Table 11:** Significance tests of partial regression coefficient and regression constant of morphological traits of crayfish cultured in aquaculture barrel.

Group	Sex	Model		Unstandardized Coefficients		Standardized Coefficients	T Value	Significant
				Regression Coefficients	Standard Error			
***D*3**	♀	1	Constant	−49.144	6.452		−7.617	0
			*X*_1_	0.518	0.043	0.938	12.084	0
		2	Constant	−34.504	4.503		−7.662	0
			*X*_1_	0.322	0.041	0.583	7.898	0
			*X*_10_	2.399	0.390	0.454	6.158	0
		3	Constant	−24.846	4.557		−5.453	0
			*X*_1_	0.052	0.085	0.094	0.610	0.549
			*X*_10_	2.998	0.356	0.568	8.419	0
			*X*_3_	0.371	0.108	0.426	3.444	0.003
		4	Constant	−22.476	2.341		−9.599	0
			*X*_10_	3.153	0.246	0.597	12.829	0
			*X*_3_	0.432	0.041	0.495	10.643	0
	♂	1	Constant	−58.344	7.878		−7.406	0
			*X*_1_	0.559	0.048	0.940	11.689	0
		2	Constant	−34.434	11.182		−3.079	0.007
			*X*_1_	0.363	0.083	0.611	4.351	0
			*X*_4_	0.669	0.249	0.378	2.691	0.015

Note: Dependent variable: body weight.

**Table 12:** Effects of morphological traits on body weight of crayfish cultured in aquaculture barrel.

Group	Sex	Trait	Correlation Coefficients	Direct Effect	Indirect Effect			Decision Coefficients
					*X*_3_	*X*_10_	Total	
***D*3**	♀	*X*_3_	0.872	0.495	—	0.377	0.377	0.618
		*X*_10_	0.910	0.597	0.313	—	0.313	0.730
	♂				*X*_1_	*X*_4_	Total	
		*X*_1_	0.940	0.611	—	0.532	0.532	0.775
		*X*_4_	0.910	0.378	0.329	—	0.329	0.545

**Table 13:** The multiple correlation analysis of morphological traits and body weight of crayfish cultured in pond.

Group	Sex	Model	Multiple *R*	Square *R*^2^	Adjusted/*R*	Standard Error	*F* Stat	Sig. *F* Stat
***D*1**	♀	1	0.909 ^A^	0.826	0.821	2.843	156.481	0.000
		2	0.955 ^B^	0.913	0.907	2.043	31.920	0.000
		3	0.967 ^C^	0.935	0.928	1.798	10.335	0.003
		4	0.973 ^D^	0.947	0.940	1.648	6.895	0.013
	♂	1	0.913 ^a^	0.833	0.825	3.299	104.668	0.000
		2	0.954 ^b^	0.909	0.900	2.489	16.871	0.001
		3	0.963 ^c^	0.927	0.916	2.285	4.742	0.042

Note: A. Predictors: (constant), cephalothorax length (*X*_5_); B. Predictors: (constant), cephalothorax length (*X*_5_), chelicerae weight (*X*_4_); C. Predictors: (constant), cephalothorax length (*X*_5_), chelicerae weight (*X*_4_), cephalothorax height (*X*_7_); D. Predictors: (constant), cephalothorax length (*X*_5_), chelicerae weight (*X*_4_), cephalothorax height (*X*_7_), caudal peduncle weight (*X*_10_). a. Predictors: constant, chelicerae weight (*X*_4_); b. Predictors: constant, chelicerae weight (*X*_4_), cephalothorax width (*X*_6_); c. Predictors: constant, chelicerae weight (*X*_4_), cephalothorax width (*X*_6_), body length (*X*_2_).

**Table 14:** The multiple correlation analysis of morphological traits and body weight of crayfish cultured in paddy field.

Group	Sex	Model	Multiple *R*	Square *R*^2^	Adjusted/*R*	Standard Error	*F* Stat	Sig. *F* Stat
***D*2**	♀	1	0.960 ^A^	0.921	0.918	1.111	257.747	0.000
		2	0.981 ^B^	0.962	0.958	0.795	21.941	0.000
		3	0.984 ^C^	0.969	0.964	0.732	4.742	0.042
		4	0.988 ^D^	0.977	0.972	0.645	6.796	0.017
	♂	1	0.977 ^a^	0.955	0.952	1.745	400.067	0.000
		2	0.986 ^b^	0.973	0.970	1.391	12.043	0.003

Note: A. Predictors: (constant), full length (*X*_1_); B. Predictors: (constant), full length (*X*_1_), caudal peduncle weight (*X*_10_); C. Predictors: (constant), full length (*X*_1_), caudal peduncle weight (*X*_10_), chelicerae weight (*X*_4_); D. Predictors: (constant), full length (*X*_1_), caudal peduncle weight (*X*_10_), chelicerae weight (*X*_4_), cephalothorax length (*X*_5_). a. Predictors: constant, cephalothorax length (*X*_5_); b. Predictors: constant, cephalothorax length (*X*_5_), chelicerae weight (*X*_4_).

**Table 15:** The multiple correlation analysis of morphological traits and body weight of crayfish cultured in aquaculture barrel.

Group	Sex	Model	Multiple *R*	Square *R*^2^	Adjusted/*R*	Standard Error	*F* Stat	Sig. *F* Stat
***D*3**	♀	1	0.938 ^A^	0.880	0.874	1.778	146.030	0.000
		2	0.980 ^B^	0.960	0.956	1.054	37.922	0.000
		3	0.988 ^C^	0.975	0.972	0.841	11.864	0.003
		4	0.988 ^D^	0.976	0.973	0.827	0.372	0.000
	♂	1	0.940 ^a^	0.884	0.877	2.869	136.637	0.000
		2	0.958 ^b^	0.918	0.909	2.472	7.240	0.015

Note: A. Predictors: constant, full length (*X*_1_); B. Predictors: constant, full length (*X*_1_), caudal peduncle weight (*X*_10_); C. Predictors: constant, full length (*X*_1_), caudal peduncle weight (*X*_10_), chelicerae length (*X*_3_); D. Predictors: constant, caudal peduncle weight (*X*_10_), chelicerae length (*X*_3_). a. Predictors: constant, full length (*X*_1_); b. Predictors: constant, full length (*X*_1_), chelicerae weight (*X*_4_).

**Table 16:** Gray correlation degree between morphological traits and body weight of crayfish cultured in pond.

Trait	Pond Cultivation Mode (D1-♀)		Pond Cultivation Mode (D1-♂)	
	Relational Order	Relational Degree	Relational Order	Relational Degree
*X*_1_	3	0.812	7	0.81
*X*_2_	6	0.793	6	0.814
*X*_3_	2	0.817	2	0.859
*X*_4_	9	0.747	10	0.756
*X*_5_	7	0.79	8	0.806
*X*_6_	4	0.802	4	0.825
*X*_7_	5	0.802	3	0.849
*X*_8_	10	0.744	9	0.761
*X*_9_	8	0.775	5	0.822
*X*_10_	1	0.855	1	0.866

**Table 17:** Gray correlation degree between morphological traits and body weight of crayfish cultured in paddy field.

Trait	Paddy Field Cultivation Mode (D2-♀)		Paddy Field Cultivation Mode (D2-♂)	
	Relational Order	Relational Degree	Relational Order	Relational Degree
*X*_1_	3	0.805	3	0.727
*X*_2_	7	0.783	9	0.694
*X*_3_	1	0.857	2	0.769
*X*_4_	9	0.724	7	0.700
*X*_5_	5	0.796	5	0.708
*X*_6_	6	0.793	4	0.723
*X*_7_	4	0.805	6	0.704
*X*_8_	10	0.717	8	0.695
*X*_9_	8	0.781	10	0.686
*X*_10_	2	0.843	1	0.877

**Table 18:** Gray correlation degree between morphological traits and body weight of crayfish cultured in aquaculture barrel.

Trait	Barrel Cultivation Mode (D3-♀)		Barrel Cultivation Mode (D3-♂)	
	Relational Order	Relational Degree	Relational Order	Relational Degree
*X*_1_	3	0.744	3	0.679
*X*_2_	5	0.73	9	0.643
*X*_3_	7	0.724	4	0.668
*X*_4_	4	0.74	2	0.707
*X*_5_	6	0.724	8	0.643
*X*_6_	2	0.746	6	0.65
*X*_7_	8	0.723	7	0.644
*X*_8_	10	0.686	10	0.632
*X*_9_	9	0.718	5	0.657
*X*_10_	1	0.801	1	0.75

## Data Availability

The data presented in this study are available in the article. Further information is available from the corresponding authors upon request.

## References

[1] Wang J.W., Yang G.L., Kong J., Xia Z.L., Sui J., Tang Q.Y., Luo K., Dai P., Meng X.H., Chen L.M. (2022). An analysis on genetic variation of threshold traits of female and male morphotypes in *Macrobrachium rosenbergii*. Oceanol. Limnol. Sin..

[2] Takata K., Nishikawa K., Otsu Y., Ui H. (2023). Intrapopulation Morphological Variation in Introduced African Clawed Frog, *Xenopus laevis* (Amphibia: Anura: Pipidae) in Japan. Curr. Herpetol..

[3] Rosilene L.D., Mayara P.N. (2020). Morphological traits correlated with resource partitioning among small characin fish species coexisting in a Neotropical river. Ecol. Freshw. Fish.

[4] Orizaola G., Laurila A. (2009). Microgeographic variation in the effects of larval temperature environment on juvenile morphology and locomotion in the pool frog. J. Zool..

[5] Dani L., Emili G.B., Francesc R.G., Cristina G., David A., Anna V.G. (2020). Captive breeding conditions decrease metabolic rates and alter morphological traits in the endangered Spanish toothcarp, *Aphanius iberus*. Int. Rev. Hydrobiol..

[6] Noëlle F., Anna V.G., Cristina G., Dolors V. (2020). Effect of environmental enrichment on the bodyshape of the pumpkinseed. Curr. Zool..

[7] Saraiva S.O., Pompeu P.S. (2014). The effect of structural enrichment in hatchery tanks on the morphology of two neotropical fish speciese. Neotrop. Ichthyol..

[8] National Aquatic Technology Promotion Station, Chinese Fisheries Society (2023). China’s Crayfish Industry Development Report (2023).

[9] Shin H.S., Montachana C.M.E., Escobar R.J.M., Álvaro L.F., Martínez S.M., María Jesús Z.S., Fernández M.J., Ramírez A.J.S., Peñate S.A., Lorenzo N.J. (2023). Genetic parameters for growth and morphological traits of the Pacific white shrimp *Penaeus vannamei* from a selective breeding programme in the industrial sector of Ecuador. Aquac. Rep..

[10] Anker A., Baeza J.A. (2012). Molecular and morphological phylogeny of hooded shrimps, genera Betaeus and Betaeopsis (Decapoda, Alpheidae): Testing the center of origin biogeographic model and evolution of life history traits. Mol. Phylogenet. Evol..

[11] Lizandra F.M., Nielson F.C.F., Caio S.N., Fernando L.M., Rogerio C.C. (2024). Advancing into the morphology of female differentiation in the seabob shrimps *Xiphopenaeus dincao* and *X. kroyeri*: Insights into the taxonomy. Reg. Stud. Mar. Sci..

[12] Lin W., Wu J.Y., Luo H.M., Liu X.L., Cao B.B., Hu F., Liu F., Yang J.F., Yang P.H. (2023). Sub-chronic ammonia exposure induces hepatopancreatic damage, oxidative stress, and immune dysfunction in red swamp crayfish (*Procambarus clarkii*). Ecotoxicol. Environ. Saf..

[13] Jiang G.M., Li J.L., Liu L., He Z.G., Zou L., Wang Y.D., Wang Q.F., Wang D.W., Deng S.M. (2023). Isolation and identification of bacterial pathogens from *Procambarus clarkii* and study on variations in serum immune factors. J. Fish. China.

[14] Xiao W.F., Hu B., Cui X.H., Cao M.X., Yao H.X., Li P., Yu L.J., Yuan H.W., Gao W.H., Tian J. (2023). Effect of egg product on growth performance, muscle nutrients, and intestinal microflora of *Procambarus clarkii*. South China Fish. Sci..

[15] Wang Q.S., Hu Q., Yang S.Q., Li Y.H. (2023). Isolation of tetrameric microsatellite markers and its application on parentage identification in *Procambarus clarkii*. Aquac. Int..

[16] Zhang L., Shi L.L., Li Y. (2019). Path analysis of morphological traits and body weight of *Procambarus clarkii*. Chin. Agric. Sci. Bull..

[17] Zhao J.L., Zhao Y.F., Song Z.C., Liu H.M., Liu Y.X., Yang R.Q. (2018). Genetic analysis of the main growth traits using random regression models in Japanese flounder (*Paralichthys olivaceus*). Aquac. Res..

[18] Yu P., Li K. (2011). Gray correlation analysis of data using Microsoft Excel. Microcomput. Appl..

[19] Zuo B.N. (2018). Effects of Shelter, Feed and Light Color on the Growth and Body Color of Red Color Juvenile Chinese Mitten Crab (Eriocheir sinensis).

[20] Weiss M., Thatje S., Heilmayer O. (2010). Temperature effects on zoeal morphometric traits and intraspecific variability in the hairy crab Cancer setosus across latitude. Helgol. Mar. Res..

[21] Zhu H.D., Gong J.H., Ma K.Y., Chen H.G., Li J.L., Feng J.B. (2022). Effects of morphological characters on body weight of wild and cultured populations of *Macrobrachium rosenbergii*. Freshw. Fish..

[22] Liu F., Chen L., Lou B., Zhan W., Chen R.Y., Xu D.D., Wang L.G., Xu L.X., Ma T., Mao G.M. (2016). Correlation and path coefficient analysis on body weight and morphometric traits of small yellow croaker *Pseudosciaena polyactis*. Oceanol. Limnol. Sin..

[23] Liu F., Chen S.L., Liu X.F., Liu Y., Cui Z.K., Deng H. (2015). Correlation and path coefficient analysis for body mass and three morphometric traits in the half-smooth tongue sole (*Cynoglossus semilaevis*). Haiyang Xuebao.

[24] Hao H.Z., Jiang H.B., Wang F., Ma H.T., Han C.H., Liu X.Q., Yang J.M. (2016). Principal component and path analysis of morphological traits of selective groups at different month ages of *Sebastes schlegeli*. J. Fish. China.

[25] Li D.Y., Pan Z., Gu Z.F., Yuan X.C., Liu X.C., Zhang M.C., Wei M., Liao X.R. (2022). Path analysis of morphological traits, body weight and pleopodal egg traits of redclaw crayfish (*Cherax quadricarinatus*). J. Hainan Trop. Ocean Univ..

[26] Huang G.H., Lei Y., Ma H.W., Feng P.F., Yang H.Z., Ruan Z.D., Huang L.M., Lv M. (2019). Comparison of parental morphology and breeding performance of different geographic populations of *Macrobrachium rosenbergii*. Fish. Sci. Technol. Inf..

